# Lightweight Low-Light Enhancement Network with Multi-Bio-Inspired Visual Mechanisms

**DOI:** 10.3390/biomimetics11060401

**Published:** 2026-06-07

**Authors:** Yafeng Zhao, Xiang Li, Shuaipeng Hao, Min Yu, Yanli Gao, Shiwei Fan

**Affiliations:** 1 College of Computer and Control Engineering, Northeast Forestry University, Harbin 150040, China; zhaoyafeng@nefu.edu.cn (Y.Z.); lx15563385385@163.com (X.L.); hsp200182@163.com (S.H.); 15621418687@163.com (M.Y.); 2 National Computer System Engineering Research Institute of China, Beijing 100083, China; 18745123763@163.com

**Keywords:** bioinspired visual perception, low-light image enhancement, lightweight neural networks, perceptual quality optimization, retinal adaptation mechanism

## Abstract

In edge deployment scenarios, low-light image enhancement faces a trade-off between model complexity and perceptual quality, limiting lightweight models under resource constraints. To address this problem, this paper proposes a perceptual quality optimization model inspired by biological visual mechanisms. Specifically, a GT-Mean loss is introduced to simulate the luminance adaptation property of the mammalian retina, effectively mitigating optimization bias caused by exposure inconsistency in imaging sensors, while the LPIPS loss, aligned with the perceptual preferences of the human visual system (HVS), is incorporated to enhance subjective visual quality. From a structural perspective, inspired by the multi-scale perception of insect compound eyes, biologically selective attention, and color constancy mechanisms, the proposed model integrates an efficient texture-aware attention module, an enhanced multi-scale feature fusion strategy, and a chrominance denoising module. Experimental results demonstrate that, while maintaining an extremely low parameter count of only 0.52 M, the proposed model consistently outperforms existing lightweight methods on the LOL series datasets in terms of PSNR, SSIM, and LPIPS. This work provides an efficient perceptual quality optimization solution for bioinspired visual sensing under resource-constrained conditions.

## 1. Introduction

Low-light image enhancement (LLIE) is a key research area in computer vision and visual sensing, aiming to improve the visual quality and usability of images captured under weak illumination. In practice, low-light images often suffer from low brightness, poor contrast, and significant noise, which not only degrade subjective perception but also impair downstream tasks such as object detection, face recognition, and autonomous driving [1,2]. Developing efficient and high-quality LLIE methods is therefore crucial. These methods can improve the stability and robustness of visual sensing systems in harsh lighting environments.

Nature has evolved diverse and efficient low-light visual systems over billions of years. These biological mechanisms offer valuable bioinspired insights for optimizing artificial visual sensing technologies. Different species adopt unique strategies to adapt to low-light environments. Flying insects like Drosophila employ non-spiking graded neuronal structures to realize efficient visual processing with low energy consumption and computational overhead [3].

Nocturnal moths are equipped with compound eyes containing special multi-scale summation units, which support high-sensitivity environmental perception in dim conditions via nonlinear spatiotemporal integration [4]. In contrast, mammals adopt various retinal adaptation mechanisms. They maintain stable luminance perception when light changes, and they adjust rod and cone cell distribution and regulate the gain of phototransduction pathways to achieve this [5]. These cross-species visual characteristics combine the high efficiency of insect vision with the perceptual adaptability of mammalian vision. Together, they provide an effective paradigm for balancing computational cost and sensing performance under resource-limited conditions. However, most existing low-light image enhancement methods do not systematically incorporate these key biological visual principles. As a result, they struggle to simultaneously achieve lightweight deployment and high-quality image restoration.

Early low-light image enhancement methods primarily relied on traditional image processing techniques. Histogram equalization (HE) enhances image contrast by redistributing pixel intensity values [6,7]; however, its global processing nature often leads to local detail loss and noise amplification, failing to exploit the cooperative local–global perception characteristics inherent in biological vision. Contrast-limited adaptive histogram equalization (CLAHE) [8] alleviates over-enhancement to some extent, yet still falls short of achieving a synergistic optimization of noise suppression and detail preservation. Methods based on Retinex theory [9,10] decompose images into reflectance and illumination components. Single-scale Retinex (SSR) [11] and multi-scale Retinex (MSR) [12] algorithms exhibit reasonable color naturalness, but their reliance on hand-crafted priors limits generalization capability, making them inadequate for adaptively handling complex low-light degradation in the manner of biological visual systems.

With the advancement of deep learning, neural network-based low-light image enhancement has become mainstream. LLNet [13] first applied deep autoencoders to enhance low-light images, achieving brightness improvement and noise suppression through end-to-end learning, yet its design relies primarily on data-driven optimization without explicitly modeling hierarchical structural and contrast perception in biological vision. RetinexNet [14] combined traditional Retinex theory with convolutional networks, decomposing images into reflectance and illumination components, partially aligning with human visual perception of lighting changes. KinD [15] and KinD++ [16] further refined this framework with improved illumination estimation and denoising, but still focused on performance optimization, paying little attention to the efficiency and low-energy characteristics of biological vision in low-light environments. MBLLEN [17] integrated multi-scale features via a multi-branch network, enhancing overall results, yet its high complexity contrasts with the streamlined efficiency of biological systems. Unsupervised and zero-reference approaches, such as Zero-DCE [18] and zero-shot latent diffusion models [19], exhibit strong generalization under limited data but lack explicit real-reference constraints, limiting structural consistency and perceptual stability for downstream vision tasks.

In recent years, Transformer architectures have introduced new advances in low-light image enhancement. SNR-Net [20] fuses local and global features through a signal-to-noise ratio (SNR)-aware mechanism, Retinexformer [21] employs an illumination-guided Transformer to model non-local interactions, and LYT-Net [22] separates luminance and chrominance in the YUV color space, achieving real-time inference with only 0.045 M parameters. However, their loss functions focus primarily on pixel-level reconstruction and neglect the optimization of perceptual quality emphasized by biological vision. As a result, they exhibit notable gaps in perceptual metrics compared with larger models, which limits their applicability in human-in-the-loop visual sensing scenarios.

Motivated by biological low-light visual mechanisms, this paper proposes a perceptual quality optimization model tailored for lightweight visual sensing applications. While maintaining extremely low model complexity, the model improves low-light image enhancement performance. It achieves this through a bioinspired loss function and optimized key modules, with particular emphasis on perceptual quality and structural detail restoration. The main contributions of this work are as follows:

- A luminance–chrominance collaborative enhancement framework is proposed for lightweight low-light image enhancement. The network separately models luminance restoration and chromatic noise suppression in the YUV space. Compared with traditional methods, this scheme avoids frequent image quality fluctuations, improving enhancement stability under complex low-light conditions.
- A bioinspired multi-scale enhancement strategy is designed to improve contextual perception and texture reconstruction. The proposed Enhanced MSEF module combines multi-scale feature extraction and texture-aware attention while maintaining low complexity compared with alternative methods.
- A lightweight perceptual optimization scheme is introduced by jointly employing GT-Mean luminance alignment and LPIPS perceptual constraints. This design improves global illumination consistency, structural fidelity, and perceptual image quality without significantly increasing model parameters, effectively eliminating uneven brightness distribution and degraded visual perception of existing methods.

To validate the practicality of the proposed model in bioinspired visual sensing scenarios, evaluations were conducted on multiple benchmark datasets. Experimental results demonstrate that, while maintaining extremely low model complexity, the method consistently improves PSNR, SSIM, and LPIPS metrics, confirming its effectiveness as a front-end enhancement module for low-light visual sensing systems, and providing technical support for reliable visual perception in complex low-light environments.

The remainder of this paper is organized as follows. Section 2 reviews related work on low-light image enhancement and bioinspired visual mechanisms. Section 3 presents the proposed lightweight enhancement model and its key modules. Section 4 provides experimental settings, comparative results, and ablation studies. Finally, Section 5 concludes the paper and discusses future research directions.

## 2. Related Work

In loss function design, Liao et al. [23] proposed GT-Mean loss to alleviate luminance mismatch during model training, which helps the network focus on structural restoration. Zhang et al. [24] developed the LPIPS metric to quantify visual similarity via deep feature distances. This metric matches well the inherent sensitivity of human vision to structural and textural features [25,26]. Regarding attention mechanisms, Hu et al.’s SE module [27] achieves feature recalibration by modeling channel dependencies, and Woo et al.’s CBAM [28] extends attention to the spatial dimension, both providing technical support for selective feature enhancement inspired by biological vision. In network design, Howard et al.’s depthwise separable convolution [29] significantly reduces computational cost, and Ronneberger et al.’s U-Net [30], with its encoder–decoder architecture and skip connections, demonstrates strong performance in denoising tasks. In other related fields, Hung et al. [31] were inspired by biological dark adaptation, and used image fusion and gamma correction in order to train deep neural networks for nighttime 2D image object detection under low-light conditions. Xiao et al. [32] proposed the ES-YOLO framework, converting RGB datasets to event-based data and integrating a hyper-redundant manipulator to detect pipeline defects in low-light environments. This work integrates these classical techniques, and introduces key biological low-light vision mechanisms, to optimize the perceptual quality of lightweight low-light enhancement models.

**Figure 1:**
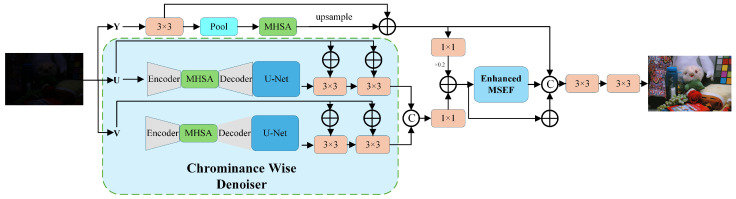
Presents the overall framework of our lightweight low-light image enhancement model based on biological visual mechanisms. The network adopts a dual-branch structure for luminance restoration and chromatic noise reduction.

Figure 1 illustrates the overall framework of the proposed model, built upon LYT-Net [22] as the baseline. The input image is decomposed into YUV components, where the Y branch employs pooling and MHSA operations to extract global luminance features, while the U and V branches are processed by the improved denoiser with MHSA and U-Net structures for chromatic noise suppression. The extracted features are fused through the Enhanced MSEF module for multi-scale feature aggregation and detail enhancement, followed by image reconstruction to generate the final enhanced result.

**Figure 2:**
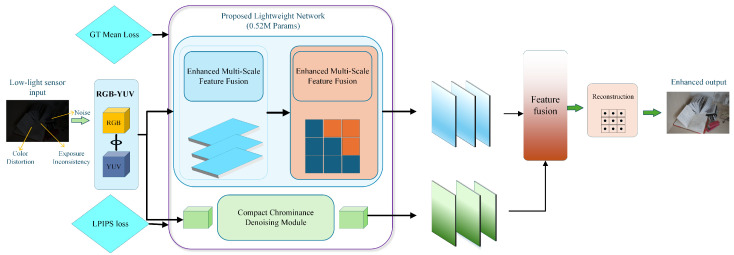
Presents the comprehensive workflow of our model.

Specifically, the model adopts a dual-path luminance–chrominance structure, analogous to the separation of luminance and color processing in biological visual systems [5], enabling more effective restoration of underexposure, noise, and color distortions in low-light sensor images. The luminance channel (Y) extracts low-level features via convolution and pooling operations, and then models global illumination relationships through a multi-head self-attention (MHSA) module, simulating the mammalian retina’s perception and adaptation to global lighting variations, in order to obtain a stable and consistent luminance representation. The chrominance channels (U, V) are denoised using the improved Chrominance-Wise Denoiser (CWD) module, which incorporates an encoder–decoder architecture combined with a soft residual output strategy. Inspired by the color constancy mechanism in biological vision, this module suppresses chrominance noise while preserving fine details and color consistency. The processed chrominance features are then fed into the Enhanced Multi-Scale Squeeze-and-Fusion (Enhanced MSEF) module, which draws on the multi-scale photoreceptor structure of moth compound eyes [4]. Parallel 3 × 3 and 5 × 5 depthwise convolutions capture fine-grained noise and coarse-grained illumination inconsistencies, respectively. An embedded lightweight texture-aware attention mechanism, inspired by selective attention in insect vision [3], adaptively enhances feature responses in textured regions, while an SE [27] module further models channel dependencies to synergistically improve perceptual quality. Finally, the fused luminance (Y) and chrominance (U, V) channels are passed through the output convolution layer to generate the enhanced image, achieving balanced luminance, color fidelity, and rich structural details for low-light visual sensing enhancement. The comprehensive workflow of the proposed model is presented in Figure 2.

## 3. Methodology

### 3.1. GT-Mean Loss Wrapper

The mammalian retina maintains stable luminance perception under changing illumination by adjusting pupil size and rod cell sensitivity. This luminance adaptation principle inspires the design of our optimization strategy. Inspired by this property, the proposed GT-Mean loss constrains global luminance consistency between enhanced images and ground-truth images, thereby reducing illumination bias. this work designs a GT-Mean loss wrapper strategy, in which the predicted image is first aligned to the mean luminance of the ground-truth image before computing the base loss, allowing for evaluation under “brightness consistency” and shifting focus to detail restoration. For any base loss function *L*, the GT-Mean-wrapped version is

(1) $$L_{GT}\left(f\left(x\right),y\right)=L\left(\hat{f}\left(x\right),y\right)$$

and the aligned prediction $\hat{f}\left(x\right)$ is calculated as

(2) $$\hat{f}\left(x\right)=clamp\left(f\left(x\right)\cdot \frac{\mu_{y}}{\mu_{f(x)}+\varepsilon },0,1\right)$$

where clamp(x,0,1) denotes a clipping operation that constrains the output to the normalized range [0,1]. Values smaller than 0 are set to 0, while values larger than 1 are set to 1. here, $\mu_{y}$ and $\mu_{f(x)}$ are the global mean luminance of the ground truth and prediction, The calculation is as follows:

(3) $$\mu_{y}=\frac{1}{CHW}\sum_{c,h,\omega }y_{c,h,\omega },\mu_{f(x)}=\frac{1}{CHW}\sum_{c,h,\omega }f{\left(x\right)}_{c,h,\omega }$$

where C,H,W denote the channel number, height, and width, respectively, and $\varepsilon =10^{-8}$ is a numerical stabilization term.

This design emulates the active luminance calibration characteristic of the biological retina. It eliminates luminance optimization bias without introducing extra parameters and exhibits good adaptability to commonly used loss functions. In this study, GT-Mean is embedded into pixel loss, perceptual loss, MS-SSIM loss, and PSNR loss to achieve effective brightness alignment.

### 3.2. LPIPS Perceptual Loss

In visual sensing tasks, relying solely on pixel-level errors often fails to capture structural distortions that may affect downstream perception modules, highlighting the need for optimization objectives aligned with human visual perception. LPIPS measures image similarity in feature space rather than pixel space by extracting multi-layer features using a pretrained deep network. Since deep features encode rich texture and semantic information, LPIPS can more effectively capture visual differences that are perceptually salient to humans. In this study, a VGG network is employed as the feature extractor. Deep features from multiple layers are first obtained for the two images, after which the weighted Euclidean distance between corresponding feature vectors is computed. Finally, the distances are aggregated across all layers and spatial locations to yield the overall perceptual distance:

(4) $$L_{lpips}=\sum_{l}\frac{1}{H_{l}W_{l}}\sum_{h,\omega }{∥\omega_{l}⊙\left(\phi_{l}\left(\hat{y}\right)-\phi_{l}\left(\hat{f}\left(x\right)\right)\right)∥}_{2}^{2}$$

$\phi_{l}$ denotes the feature extraction at the *l* layer of the VGG network, while $H_{l},W_{l}$ represent the height and width of the feature map at that layer, and is the channel weight vector. Note that the input images must be normalized to the range [−1,1], The total loss function is defined as:

(5) $$\hat{y}=2y-1,\hat{f}\left(x\right)=2f\left(x\right)-1$$

It is worth noting that the LPIPS loss is not wrapped with GT-Mean. This is because deep features are inherently robust to global brightness variations and are more sensitive to local texture and structural differences. LPIPS thus complements the pixel-level losses wrapped by GT-Mean: the latter eliminates luminance bias to encourage detail restoration, while the former provides optimization guidance directly at the perceptual level. The total loss function is defined as

(6) $$L_{total}=\alpha_{1}L_{GT}^{pixel}+\alpha_{2}L_{GT}^{perc}+\alpha_{3}L_{GT}^{ssim}+\alpha_{4}L_{GT}^{psnr}+\alpha_{5}L_{lpips}+\alpha_{6}L_{hist}+\alpha_{7}L_{color}$$

where $\alpha_{1}L_{GT}^{pixel},\alpha_{2}L_{GT}^{perc},\alpha_{3}L_{GT}^{ssim}$ and $\alpha_{4}L_{GT}^{psnr}$ denote the pixel, perceptual, MS-SSIM, and PSNR losses wrapped by GT-Mean, respectively. $\alpha_{5}L_{lpips}$ represents the LPIPS perceptual loss, $\alpha_{6}L_{hist}$ is the histogram loss used for matching luminance distribution, and $\alpha_{7}L_{color}$ is the color loss utilized to maintain color consistency. All weighting coefficients $\alpha_{1}-\alpha_{7}$ are set as positive empirical hyperparameters to maintain normal gradient descent and balance the contribution of each loss branch. Importantly, these weights are not constrained to sum to one. Since each individual loss term corresponds to different optimization dimensions including pixel reconstruction, luminance alignment, perceptual similarity, histogram distribution and color consistency, their numerical magnitudes are naturally inconsistent. Imposing a sum-to-one constraint will unreasonably suppress or amplify certain loss items, destroying the joint supervision effect of the multi-loss framework.

### 3.3. Texture Attention Mechanism

**Figure 3:**
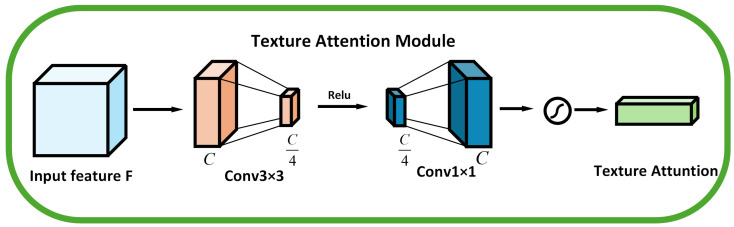
Framework of the Texture Attention mechanism.

Under low-light conditions, texture regions in images captured by visual sensors are more susceptible to noise and underexposure, necessitating selective feature enhancement for textured structures. To address this, a lightweight texture-aware attention mechanism is designed. Inspired by the selective attention principle in insect vision, biological visual systems selectively emphasize informative regions while suppressing redundant background information. Leveraging this biological property, the proposed Texture Attention module generates spatial attention weights to enhance texture-rich regions and preserve structural details during image enhancement, which prioritizes texture-rich regions while suppressing irrelevant noise. The structure of this mechanism is illustrated in Figure 3. It adaptively enhances feature responses in texture regions by learning a spatial attention map. This mechanism generates pixel-wise attention weights in the spatial dimension, employs a channel compression strategy to reduce computational overhead, utilizes 3×3 convolutions to capture local texture patterns, and generates weights in the range [0,1] via a Sigmoid function for smooth modulation. Given input features $F\in R^{C\times H\times W}$, the channel count is first compressed to one-fourth using a convolution while extracting local texture features:

(7) $$F_{compress}=\delta \left(W_{1}*F\right)$$

where $W_{1}\in R^{C\times \frac{C}{4}\times 3\times 3}$ denotes the convolution kernel and δ is the ReLU activation function. Subsequently, a 1×1 convolution restores the channel dimension and generates the attention map:

(8) $$A=\sigma (W_{2}*F_{compress})$$

where $W_{2}\in R^{C\times \frac{C}{4}\times 1\times 1}$ is the convolution kernel, σ is the Sigmoid activation function, and $A\in R^{C\times H\times W}$ is the generated spatial attention map. Finally, the attention map is element-wise multiplied with the input features to achieve feature recalibration:

(9) $$F^{\prime }=F⊙A$$

This design enables the network to automatically learn which spatial locations contain significant texture information and enhance the feature response of these regions via attention weighting, thereby better preserving detailed textures during the enhancement process.

### 3.4. Multi-Scale Feature Extraction Module

**Figure 4:**
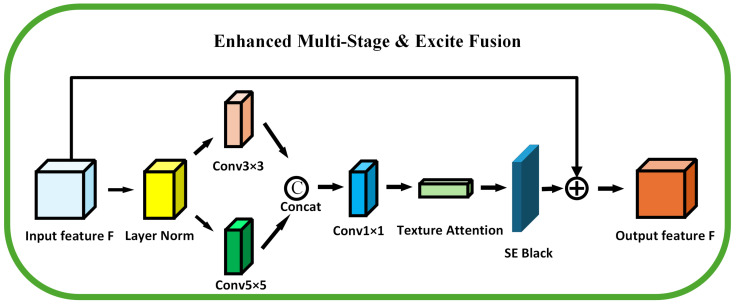
Multi-scale feature extraction module.

The degradation of low-light images typically exhibits multi-scale characteristics: fine-grained noise requires processing with a small receptive field, whereas large-scale illumination nonuniformity necessitates a larger receptive field. Such multi-scale degradation is particularly pronounced in real-world visual sensing. To address this, an Enhanced Multi-Scale Squeeze-and-Fusion (Enhanced MSEF) module is designed, as illustrated in Figure 4. Biologically, compound eyes consist of multiple ommatidia that perceive local regions independently. These local observations are further integrated into global visual perception. Inspired by this mechanism, the proposed module adopts parallel multi-scale convolution branches, so as to simulate distributed receptive-field perception and contextual feature aggregation under low-light conditions. The module employs parallel depthwise convolutions with 3 × 3 and 5 × 5 kernels to extract fine- and coarse-grained features, respectively. Input features are first processed through layer normalization and then fed into the two depthwise convolution branches corresponding to the different scales:

(10) $$F_{3\times 3}=DWConv_{3\times 3}\left(LN\left(F_{in}\right)\right),F_{5\times 5}=DWConv_{5\times 5}\left(LN\left(F_{in}\right)\right)$$

where $F_{in}\in R^{C\times H\times W}$ is the input feature, LN denotes the layer normalization operation used for training stability, and DWConv represents depthwise convolution (where the number of groups equals the number of channels), which significantly reduces computation compared to standard convolution. The features from both scales are concatenated along the channel dimension and fused via a 1×1 pointwise convolution:

(11) $$F_{multi}=Conv_{1\times 1}\left(Concat\left(F_{3\times 3},F_{5\times 5}\right)\right)$$

The fused multi-scale features are sequentially enhanced by the Texture Attention module and the SE Channel Attention module. Texture Attention identifies important texture regions in the spatial dimension, while SE Attention models inter-channel dependencies; together, they synergistically model “where is important” spatially and “what is important” channel-wise. Finally, a residual connection is employed to ensure gradient flow.

(12) $$F_{out}=SE\left(TextureAttn\left(F_{multi}\right)\right)+F_{in}$$

### 3.5. Chroma Denoising Module

Based on the principle of biological color constancy, the visual system can maintain stable color perception under illumination variations and visual noise interference. Inspired by this characteristic, we design a compact chroma denoising module. It separates chrominance restoration from luminance enhancement to stabilize object colors, balance noise suppression and detail preservation, and suppress chromatic noise in low-light images. In low-light visual sensing scenarios, chrominance channels are generally more susceptible to sensor noise and quantization errors than the luminance channel. Therefore, this module employs a U-Net-based encoder–decoder architecture specifically to suppress noise in the chrominance channels, maintaining color constancy while avoiding detail loss, as illustrated in Figure 1. It preserves texture details through the skip connections of U-Net and incorporates multi-head self-attention at the bottleneck layer to capture global contextual information. The encoder consists of four convolutional layers that progressively downsample the feature maps to extract multi-scale features:

(13) $$E_{i}=\delta \left(Conv_{↓}\left(E_{i-1}\right)\right),i=1,2,3,4$$

where $E_{0}$ is the input single-channel chroma image, $Conv_{↓}$ denotes a downsampling convolution with a stride of 2, and δ is the ReLU activation function. Through progressive downsampling, the encoder expands the receptive field while extracting multi-scale feature representations ranging from low-level textures to high-level semantics. The bottleneck employs a multi-head self-attention (MHSA) mechanism to capture global dependencies:

(14) $$B=MHSA(E_{4})$$

The self-attention mechanism enables the network to model correlations between any two spatial positions, effectively capturing the global structural information of the image, which is particularly critical for handling large-scale color noise. The decoder restores spatial resolution via progressive upsampling with transposed convolutions, and concatenates features with corresponding encoder layers to build skip connections:

(15) $$D_{i}=\delta (Conv(Concat\left(Up\left(D_{i+1}\right),E_{i}\right),i=3,2,1$$

where $D_{4}=B$ is the bottleneck output and $U_{p}$ denotes the transposed convolution upsampling operation. Skip connections directly transfer detail information from the encoder to the decoder, compensating for spatial details lost during downsampling and ensuring that the denoised image retains clear texture edges. To avoid the loss of original details caused by excessive denoising, we adopt a soft residual output strategy:

(16) $$I_{out}=I_{in}+\beta \cdot D_{1}$$

where $I_{in}$ is the input chroma image, $D_{1}$ is the output of the denoising module, and β=0.5 is the soft residual coefficient.

To further clarify the relationship between the adopted biological inspiration mechanisms and the proposed architectural components, the corresponding biological principles and network modules are summarized in Table 1.

Compared to directly outputting the denoised result, the soft residual connection fuses denoised information with lower weight, preserving original textures while effectively suppressing noise.

## 4. Experiments and Discussion

### 4.1. Implementation Details

The proposed model is implemented using the PyTorch (2.12.0) framework and trained on a single NVIDIA RTX 4090 GPU (manufactured by NVIDIA Corporation in Santa Clara, CA, USA) with CUDA acceleration. The AdamW optimizer is adopted for network optimization with $\beta_{1}=0.9$, $\beta_{2}=0.999$, and a weight decay of $1\times 10^{-4}$. The initial learning rate is set to $1\times 10^{-4}$.

A warmup and cosine annealing learning rate strategy is employed during training. Specifically, the learning rate is linearly increased from 0 during the first 50 epochs and then gradually decayed to $1\times 10^{-6}$ using cosine annealing.

The proposed network is trained for 2000 epochs with a batch size of 1. During training, image pairs are randomly cropped into 256×256 patches. Random horizontal flipping and random rotation are adopted for data augmentation. Gradient clipping with a maximum norm of 1.0 is further employed to stabilize the training process.

The model is trained and evaluated on LOLv1, LOLv2-R, and LOLv2-S datasets, with train:test splits of 458:15, 689:100, and 900:100, respectively. Model validation is performed after each training epoch.

The ImprovedLYT architecture employs 32 channels as the base feature dimension. For fair comparison, all evaluation metrics, including PSNR, SSIM, and LPIPS, are computed under the same evaluation protocol. Specifically, GT-Mean alignment is adopted for PSNR/SSIM evaluation, while LPIPS is calculated using the AlexNet backbone (from the official LPIPS package with pre-trained ImageNet weights).

For data preprocessing, training image pairs are randomly cropped into 256×256 patches. Random horizontal flipping and random rotation are applied for data augmentation to mitigate overfitting. The batch size is set to 2, and training is conducted for 2000 epochs. PSNR, SSIM, and LPIPS are used as evaluation metrics.

### 4.2. Comparative Study

Quantitative Results: We compare the proposed model with existing lightweight methods with a focus on performance and complexity on the LOL dataset. As shown in Table 2, sll metrics are computed after GT-Mean correction. ↑ indicates that higher values are preferable, whereas ↓ indicates that lower values are preferable. The best results are marked in red, and the second-best in blue. In this work, we strictly focus on lightweight edge-oriented low-light enhancement tasks, so the selection of comparison methods follows the principle of consistent application scenarios and model lightweight constraints. For fair comparison, all baseline methods except LYT-NET are evaluated using their official pretrained models. LYT-NET is re-trained using its official code under the same training settings as our method. All models share the same dataset, image size and format during training and testing. Some other mainstream low-light image enhancement models may achieve better results; however, most of these superior methods come with extremely large parameter sizes ranging from 114 M [33] to 1252 M [34], accompanied by heavy computational overhead. Such large-scale models are primarily designed for high-performance workstations and cannot be deployed on lightweight edge devices with limited memory and computing resources. In contrast, our method is specially tailored for resource-constrained visual sensing scenarios. Therefore, we mainly select representative lightweight algorithms for fair comparison, while still listing several classic large-scale methods in Table for intuitive reference and comprehensive comparison. All methods are evaluated using identical input resolutions and dataset protocols. Our model contains only 0.52 M parameters, offering a lightweight design suitable for resource-constrained visual sensing systems. Experimental results show that the proposed method integrates bioinspired luminance adaptation, structural perception and multi-scale feature aggregation. It achieves an excellent balance between lightweight deployment, visual perceptual quality and restoration fidelity under limited computational resources. This optimization scheme effectively meets the requirements of front-end enhancement in visual sensing systems, providing efficient and accurate low-light image processing capabilities. Moreover, the quantitative results combined with subsequent multi-criteria ranking further validate the competitiveness of our method in comprehensive performance.

**Figure 5:**
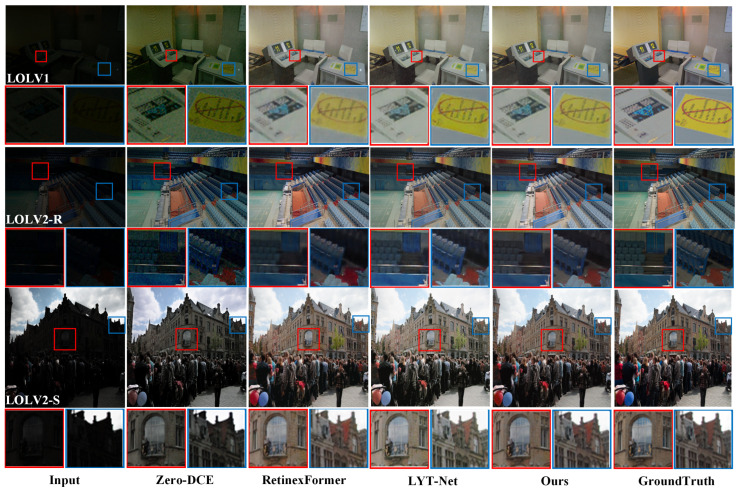
Qualitative comparison of LLIE methods on the LOL dataset, with magnified regions highlighting the differences.

Qualitative Results: The qualitative performance of our model compared with other LLIE methods is shown in Figure 5. For comparison, LYT-Net results are obtained under the same training environment as our model. Supervised methods such as Zero-DCE [18] and Zero-DCE++ [35] exhibit strong adaptability to dark conditions, but their performance in specific scenes remains inferior to other supervised models. RetinexFormer [21] achieves notable progress in low-light restoration, yet suffers from issues such as texture blurring. LYT-Net [22] reaches a very high overall performance but neglects structural perception. Overall, our proposed model demonstrates highly effective performance through the synergistic action of the bioinspired modules.

### 4.3. Multi-Criteria Analysis

To provide a comprehensive comparison across different evaluation metrics, a normalized multi-criteria analysis is conducted based on the averaged results over the LOLv1, LOLv2-R, and LOLv2-S datasets, including PSNR, SSIM, LPIPS, and model parameters. For each method, the metric values obtained on the three datasets are first averaged to reduce dataset-specific bias and provide a more reliable overall evaluation.

For metrics with higher values indicating better performance, the normalized score is computed as

(17) $$M_{i}^{norm}=\frac{M_{i}-M_{min}}{M_{max}-M_{min}}$$

For metrics with lower values indicating better performance, the inverse normalization is adopted:

(18) $$M_{i}^{norm}=\frac{M_{max}-M_{i}}{M_{max}-M_{min}}$$

The final comprehensive score is calculated as

(19) $$Score=\frac{1}{N}\sum_{i=1}^{N}M_{i}^{norm}$$

where *N* denotes the number of evaluation metrics, including PSNR, SSIM, LPIPS, and model parameters. Equal weights are adopted to avoid introducing subjective bias in the evaluation process.

As shown in Table 3, the proposed method achieves the highest overall ranking among the compared lightweight LLIE approaches. Although several large-scale models obtain competitive results on individual metrics, their substantially higher parameter complexity limits their applicability in resource-constrained visual sensing scenarios.

Compared with LYT-NET, the proposed method achieves consistently better PSNR, SSIM, and LPIPS performance across multiple datasets while maintaining relatively low model complexity. This demonstrates that the introduced bioinspired mechanisms effectively improve structural restoration and perceptual fidelity without introducing excessive computational overhead.

Overall, the proposed model provides a balanced trade-off between enhancement quality, perceptual consistency, and lightweight deployment efficiency, demonstrating its suitability for edge-oriented low-light visual sensing applications.

**Figure 6:**
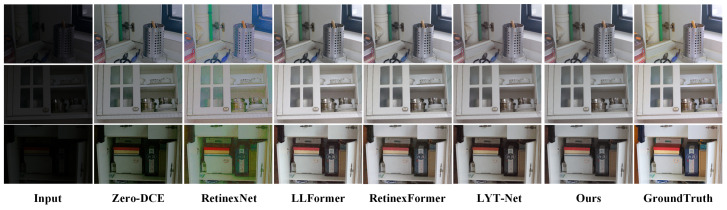
Qualitative comparison of LLIE methods on the LOLv1 dataset.

**Figure 7:**
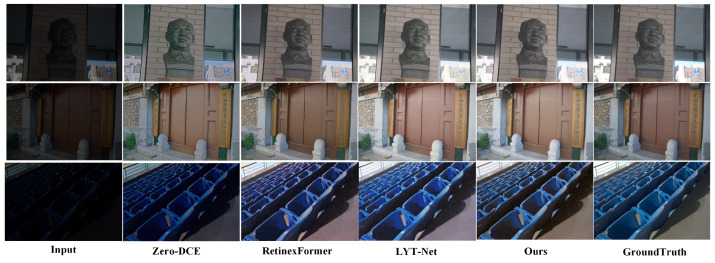
Qualitative comparison of LLIE methods on the LOLv2-R dataset.

**Figure 8:**
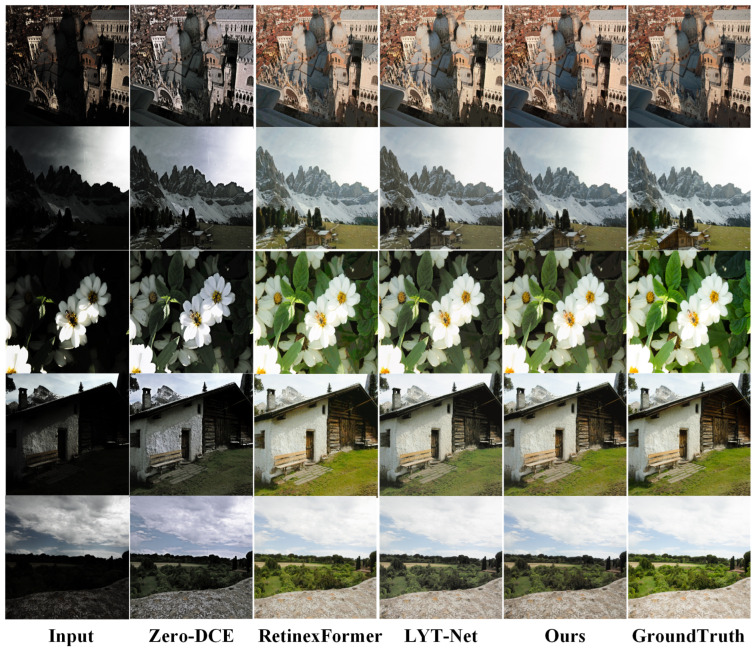
Qualitative comparison of LLIE methods on the LOLv2-S dataset.

To better demonstrate the overall performance of our model across multiple datasets, we conduct qualitative comparisons on multiple images from the LOLv1, LOLv2-Real Captured, and LOLv2-Synthetic datasets against other LLIE methods. The comparative results are illustrated in Figure 6, Figure 7 and Figure 8.

These overall visual comparison experiments highlight the advantages of our model in color restoration and detail preservation, demonstrating its effectiveness in low-light image enhancement. At the same time, the model achieves excellent denoising performance and produces results more consistent with human visual perception. Furthermore, the PSNR of our model on the LOLv2-S dataset is significantly higher than that of LYT-Net, further indicating its strong potential on synthetic data.

### 4.4. Unpaired Datasets Experiments

**Figure 9:**
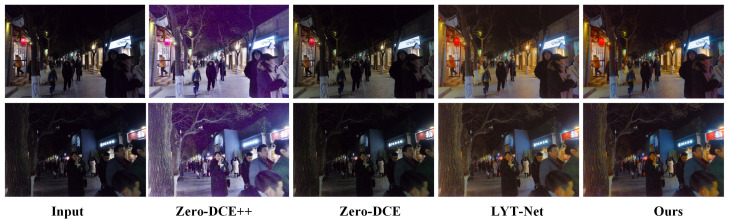
Qualitative comparison of LLIE methods on the Dark-face dataset.

To evaluate the visual quality of restored images, we randomly select 100 real low-light samples from the Dark-face dataset for testing. We use the official pretrained models for Zero-DCE and Zero-DCE++, and other models adopt weights trained on the LOLv2-S dataset, as mentioned before. We adopt two no-reference image quality metrics, NIQE [39] and BRISQUE [40]. ↓ indicates that lower values are preferable. The quantitative results are listed in Table 4, with the best results marked in red, and visual comparisons are presented in Figure 9. The experimental results show that our method outperforms other algorithms and achieves remarkable improvements on both metrics.

### 4.5. Ablation Study

On the LOLv1, LOLv2-R, and LOLv2-S datasets, we conducted systematic ablation studies using model complexity, PSNR, SSIM, and LPIPS as quantitative evaluation metrics to analyze the individual contributions of the five proposed components. The ablation results on the three datasets are reported in Table 5, Table 6 and Table 7. The best results are highlighted in bold.

The results show that introducing GT-Mean loss and LPIPS loss significantly improves performance across multiple enhancement metrics without increasing model complexity, enhancing brightness consistency and perceptual stability. The designed Texture Attention mechanism effectively boosts PSNR, indicating its positive role in restoring texture structures. Meanwhile, the Enhanced MSEF and Improved Denoiser contribute substantially to overall enhancement quality, albeit with a modest increase in model complexity.

## 5. Conclusions

This work addresses the fundamental challenge in low-light visual sensing scenarios, where lightweight enhancement models struggle to balance perceptual quality and structural detail restoration. We propose a bioinspired, low-complexity model for perceptual quality optimization. The model incorporates a GT-Mean loss that simulates the adaptive mechanisms of the biological retina, effectively mitigating optimization bias caused by sensor exposure inconsistencies. In combination with the LPIPS perceptual loss, which aligns with the human visual system’s inherent preference for structural and textural features, the model maintains strong structural and texture awareness under lightweight constraints. Furthermore, by drawing on multi-scale perception and selective attention mechanisms of insect compound eyes, we design a texture-aware attention module, an enhanced multi-scale feature fusion module, and a compact chrominance denoising module that simulates biological color constancy, collectively improving detail fidelity and color consistency. Experimental results on multiple benchmark low-light datasets, including LOLv1, LOLv2-Real, and LOLv2-Synthetic, demonstrate that the proposed method outperforms existing lightweight approaches in key metrics such as PSNR, SSIM, and LPIPS, while requiring only 0.52 M parameters, making it well-suited for resource-constrained visual sensing and edge computing scenarios.

Although the proposed method achieves a good trade-off between visual performance and lightweight deployment, it still has several limitations. Our model is mainly tested on the LOL series datasets, which fail to cover diverse real-world low-light conditions like extreme darkness, motion blur and bad weather. In addition, our approach relies heavily on paired low-light and normal-light images. Unpaired data will prevent the model from learning effective brightness and texture transformation, leading to poor enhancement and unstable outputs. The bioinspired modules improve visual quality, yet multi-scale attention and other components add extra computational cost compared with ultra-lightweight models such as LYT-Net. In addition, the GT-Mean strategy works well for global luminance adjustment but is less capable of handling severe local illumination inconsistency. Finally, this framework is built solely for image enhancement and has not been applied to downstream tasks including low-light object detection and segmentation.

Future work will improve the generalization ability of the proposed model in complex real-world low-light environments. We will also explore more efficient lightweight attention mechanisms to further reduce computational cost. In addition, the proposed framework will be extended to video low-light enhancement and downstream vision tasks, such as object detection and autonomous visual sensing.

## Figures and Tables

**Table 1:** Correspondence between biological inspiration mechanisms and network modules.

Biological Mechanism	Corresponding Module	Core Function
Luminance adaptation mechanism	GT-Mean Wrapper	Global luminance consistency alignment
Visual perceptual similarity mechanism	LPIPS Perceptual Loss	Perceptual feature consistency preservation
Selective attention mechanism	Texture Attention Mechanism	Adaptive enhancement of texture-rich regions and structural details
Compound eye perception	Multi-Scale Feature Extraction Module	Multi-scale feature extraction and contextual aggregation
Color constancy mechanism	Chroma Denoising Module	Chromatic noise suppression and stable color reconstruction

**Table 2:** Quantitative comparison on the LOL dataset.

Methods	Params/M	LOLv1			LOLv2-R			LOLv2-S		
		PSNR ↑	SSIM ↑	LPIPS ↓	PSNR ↑	SSIM ↑	LPIPS ↓	PSNR ↑	SSIM ↑	LPIPS ↓
RetinexNet [14]	0.84	18.915	0.427	0.470	18.323	0.447	0.519	19.099	0.774	0.247
ZeroDCE [7]	0.075	20.050	0.535	0.353	21.463	0.540	0.350	21.463	0.848	0.149
ZeroDCE++ [35]	0.01	18.516	0.434	0.360	18.659	0.371	0.356	19.834	0.837	0.180
LLFlow [36]	17.42	24.998	0.871	0.117	25.421	0.877	0.158	27.961	0.930	0.063
LED [37]	7.07	25.470	0.846	0.113	27.814	0.870	0.114	27.367	0.928	0.056
LLFormer [38]	24.55	25.758	0.823	0.117	26.197	0.819	0.209	28.006	0.927	0.061
RetinexFormer [21]	1.53	27.140	0.850	0.129	27.694	0.856	0.166	28.992	0.939	0.056
LYT-NET	0.05	25.800	0.837	0.136	28.013	0.876	0.124	26.646	0.926	0.071
Ours	0.52	26.495	0.854	0.108	28.475	0.884	0.114	28.384	0.938	0.054

**Table 3:** Normalized multi-criteria ranking results on the LOL datasets.

Method	Overall Score	Rank
Ours	0.990	1
RetinexFormer [21]	0.957	2
LYT-NET	0.945	3
LED [37]	0.889	4
LLFlow [36]	0.757	5
LLFormer [38]	0.660	6
ZeroDCE [7]	0.478	7
ZeroDCE++ [35]	0.345	8
RetinexNet [14]	0.243	9

**Table 4:** Quantitative comparison of NIQE and BRISQUE on Dark-face images.

Model	NIQE ↓	BRISQUE ↓
Zero-DCE++	3.623	37.736
Zero-DCE	3.447	26.822
LYT	2.846	14.484
Ours	2.807	13.342

**Table 5:** Ablation study on the LOLv1 dataset.

Metrics	Params/M	PSNR ↑	SSIM ↑	LPIPS ↓
NO GT-Mean loss	0.52	26.000	0.847	0.119
NO LPIPS loss	0.52	26.241	**0.854**	0.116
NO Texture Attention	0.50	26.363	**0.854**	0.109
NO Enhanced MSEF	0.47	26.066	0.848	0.119
NO improved Denoiser	0.38	26.161	0.849	0.117
Full Model	0.52	**26.495**	**0.854**	**0.108**

**Table 6:** Ablation study on the LOLv2-R dataset.

Metrics	Params/M	PSNR ↑	SSIM ↑	LPIPS ↓
NO GT-Mean loss	0.52	27.920	0.873	0.126
NO LPIPS loss	0.52	28.185	0.883	0.122
NO Texture Attention	0.50	28.314	**0.884**	0.116
NO Enhanced MSEF	0.47	28.022	0.874	0.125
NO improved Denoiser	0.38	28.037	0.876	0.123
Full Model	0.52	**28.475**	**0.884**	**0.114**

**Table 7:** Ablation study on the LOLv2-S dataset.

Metrics	Params/M	PSNR ↑	SSIM ↑	LPIPS ↓
NO GT-Mean loss	0.52	28.016	0.932	0.062
NO LPIPS loss	0.52	28.192	0.937	0.059
NO Texture Attention	0.50	28.277	**0.938**	0.056
NO Enhanced MSEF	0.47	28.053	0.933	0.061
NO improved Denoiser	0.38	28.110	0.934	0.060
Full Model	0.52	**28.384**	**0.938**	**0.054**

## Data Availability

The public datasets used in this paper and their official access links are as follows: LOLv1: https://daooshee.github.io/BMVC2018website/ (accessed on 15 November 2025) LOLv2-real and LOLv2-synthetic: https://huggingface.co/okhater (accessed on 15 November 2025) Dark-face: https://flyywh.github.io/CVPRW2019LowLight/ (accessed on 27 May 2026).
